# Effect of social support on Muslim women’s sporting activities: mediating effect of psychological adjustment

**DOI:** 10.3389/fpsyg.2024.1335886

**Published:** 2024-09-04

**Authors:** Nan Chen, Fengjie Qiao

**Affiliations:** ^1^Anyang Institute of Technology, Anyang, China; ^2^Department of Physical Education, Tsinghua University, Beijing, China

**Keywords:** Muslim women, social support, psychological adjustment, sporting activities, effect mediation

## Abstract

**Objective:**

This study explores the relationship between social support and sporting activities of Muslim women and constructs a mediation model through role of psychological adjustment.

**Methods:**

Using stratified cluster sampling, 301 Muslim women were measured in group psychology using the Social Support Scale and the Sports Activities and Psychological Adjustment Scale. The statistical software SPSS 24.0 and SPSS PROCESS 3.3 were used for statistical processing. The common-method variation test was carried out using the Harman single-factor control test. Finally, the Bootstrap sampling test method and process plug-in were used to test the significance of the intermediary effect.

**Results:**

(1) Social support has a significant predictive effect on sports activities (*β* = 0.32, *p* < 0.001); (2) psychological adjustment (*β* = 0.552, *p* < 0.001) mediates the relationship between social support and sporting activities [social support → psychological adjustment → sporting activities (95% Cl, 0.093, 0.323)].

**Conclusion:**

Social support positively influences sporting participation among Muslim women, and psychological adjustment mediates this relationship. Strengthening social support for Muslim women can enhance their psychological adjustment, thereby improving their participation in sporting activities and offering valuable theoretical and practical guidance.

## 1 Introduction

The promulgated Outline of Building a Sports Powerful Country highlights the necessity to improve the physical quality of the whole nation, particularly the promotion of sports activities and physical health interventions for women and other groups (Huang, 2024). However, lingering traditional beliefs and societal norms perpetuate sexist attitudes in sports (Xiong et al., 2022). The prevailing traditional notion of male superiority and the responsibilities of domestic life exert pressure on Muslim women with religious beliefs, which can lead to a lack of engagement in sports activities (Salma et al., 2020; Thorpe Ahmad et al., 2022). The physical conditions and living habits of women can influence their ability to support their development and affect their physical health and living habits, as well as influence the next generation (Xiong, 2021).

Female emancipation and the fight for women’s rights have resulted in female sporting participation becoming a focus of research in the international sports field. Research on women’s physical activity in China began with the localization of Western feminism in the 1980s; however, there is relatively little research on the physical activity of Muslim women. The motivation of Muslim women to participate in sports not only stems from their upbringing but is also a result of their resistance to traditional gender culture and institutions. The motivation for physical activity for Muslim women is constantly evolving and changing, and the mechanisms that produce these changes are complex, understanding the specific and nuanced individual experiences of Muslim women with physical activity and the facts behind these individual experiences is a useful addition to the current research. As a result, this study adopted an empirical research paradigm based on the Social Ecological Theory and Social Cognitive Theory (SCT) to analyze the causes of “social support” and the “psychological adaptation” of physical activity in Muslim women at a micro level. Further, this study aimed to determine how social support affects the sporting activities of Muslim women to add to the existing literature and support practical operations. This is especially important in the context of China because Muslim women in China have experienced sociocultural changes as a result of “sports power policies” in this nation. By comprehensively studying the interplay between these factors, we can enhance our understanding of the sporting activities of Muslim women.

There are mainly two reasons for Muslim women’s lack of sporting participation. First, cultural and religious practices: many Muslim communities have strict norms and beliefs about modesty and the appropriate public behavior for women (Jarvie et al., 2013). These can limit the participation of women in public physical activities. The requirement to wear traditional clothing that covers the body, such as hijabs and abayas, can also be a barrier to participating in sports or exercise, especially in environments that do not accommodate these dress codes (Summers et al., 2018). Second, lack of social support: there is often a shortage of sports facilities that cater to the privacy and specific needs of Muslim women (Mai and Shi, 2019). Without access to gender-segregated gyms or women-only exercise classes, many Muslim women may feel uncomfortable or unable to participate in physical activities, which also reduces their opportunities for exercise (Toffoletti and Palmer, 2017). Promoting female health requires support, education at the social group and individual levels to make sports part of a healthy female lifestyle (Xiong, 2021). Social support is an essential factor in promoting the maintenance and development of female sports participation (Dong, 2017).

The Social Support Theory refers to individual support at practical and emotional levels through social connection, which provides a sense of security, belonging, and self-value (Xu, 2021). The Social Ecological Theory can be effectively integrated with social support concepts to comprehensively understand how social environments influence individual behavior and health outcomes. The Social Ecological Theory has three levels (1) Microsystem: At this level, social support is typically provided through immediate relationships, such as family, friends, peers, and colleagues. These support networks are crucial for promoting healthy behaviors and providing emotional, educational, and practical support (Piel et al., 2017). For example, family members can encourage regular physical activity or a balanced diet. (2) Mesosystem: The interactions between the different microsystems can enhance or undermine the effectiveness of social support. For instance, supportive relationships at home that are reinforced by similar support at school or work can strengthen overall well-being (Devereux et al., 2015). (3) Exosystem: Although individuals may not directly interact with the exosystem, it can affect the types of social support they receive. For example, community resources, such as clubs, religious organizations, and social services, can also provide critical social support even if the individual is not directly involved with them (Scharpf et al., 2021).

In sports research, the Social Ecological Theory examines the context in which individuals engage in physical activity, emphasizing the impact of various factors at different levels on their exercise habits (Martins et al., 2017). The theory recognizes that an individual’s exercise behavior is influenced by both internal and external factors (Tang, 2018). Domestic and foreign studies show that social support positively influences sporting participation (Pan et al., 2020; Lin and Xiong, 2021; Hui et al., 2022). Support from material and financial resources, as well as emotional encouragement from family and friends, is crucial for fostering and maintaining engagement in sports and exercise (Cho et al., 2023). Psychological adjustment involves the use of psychological science techniques to help individuals and others achieve a healthy mental state. Some researchers have recognized that psychological adjustment is reflected in psychological resilience (Mu et al., 2019). This positive personality trait can reduce the negative influence of external adverse factors and hazardous factors on the psychological and developmental adaptation of the individual. Generally, actively practicing psychological adjustment fosters happiness and provides the foundation for success. Mastering psychological adjustment can heighten self-awareness, fortify interpersonal connections, reduce personal stress, and enhance adaptability (Wang et al., 2020).

The SCT is commonly used to explain trinities and human behavior in the reciprocal model, indicating that the mutual effect of humans and nature can determine individual behavior. The environment plays a crucial role in shaping an individual’s ability to self-regulate, thus fostering and developing the ability of self-regulation (Schunk and DiBenedetto, 2020). This implies that cognitive, environmental, and behavioral factors can influence the individual’s actions (Liu and Wang, 2023). Therefore, the effects of social support and psychological adjustment should be considered when studying the influencing mechanism of the sporting activities of Muslim women. SCT and social support concepts interact when understanding and influencing human behavior, particularly in the context of health behaviors and interventions (Bashirian et al., 2021). SCT emphasizes the importance of observational learning, self-efficacy, and the dynamic interaction between a person, their behavior, and their environment.

Social support provides opportunities for observational learning, where individuals can learn by watching others successfully manage similar challenges. For instance, observing a family member or friend effectively manage a healthy diet or a consistent exercise routine can inspire and teach others within the social network to emulate those behaviors (Luszczynska and Schwarzer, 2020). Social support has a direct impact on an individual’s belief in their ability to succeed in specific situations (self-efficacy). Positive reinforcement from peers, family, or mentors increases confidence in one’s abilities, which makes it more likely that the individual will engage in and maintain healthy behaviors. Supportive feedback, encouragement, and shared experiences contribute to higher self-efficacy (Ogbe et al., 2020). This principle of SCT states that a person’s behavior, personal factors (such as cognitions and emotions), and environmental influences all interact and influence each other. Social support is a crucial environmental factor that can alter personal beliefs and, consequently, behaviors. For example, a supportive social network can help individuals quit smoking or adhere to medication regimes by providing emotional support, practical help, and positive social norms (Neneh, 2022).

Social support also influences the expected outcomes from engaging in certain behaviors. If the social environment is supportive, individuals might expect more positive outcomes from their behavior changes, such as greater enjoyment in physical activities or better stress management. These positive expectations can motivate continued behavior change (Chen et al., 2020). Previous research has shown that many health promotion programs use SCT to design strategies incorporating social support mechanisms to enhance their effectiveness. Programs that involve group activities or peer-led interventions can increase participation and success by harnessing the motivational and instructional aspects of social support (Marcionetti and Castelli, 2023). In summary, the combination of SCT and Social Support Theory provides a robust framework for understanding and influencing behavior change. Social support enriches the SCT framework by adding a layer of community and interpersonal influences, which can significantly enhance the effectiveness of behavior change interventions. This integration is particularly beneficial in health-related behaviors where both psychological and social factors play crucial roles.

During the critical period of the all-round implementation of the Outline of Country Revitalization through Sports, research on the impact of social support on the sporting activities of Muslim women and an understanding of the mechanisms and effects involved have become urgent priorities. Previous research suggests that the literature on Muslim women and sports activities primarily focuses on the barriers to participation in sports activities and potential solutions (Dagkas and Benn, 2006; Kay, 2006). Cultures and customs differences between Muslim religious sects and communities are notably significant (Tammy and Rail, 2005), and there is limited research on the participation of Muslim women in sports activities in central China. To this end, this study delved into the relationship between social support and the engagement of Muslim women in sports activities in central China based on the SCT and Social Ecological Theory.

To the best of our knowledge, there have been no studies that have assessed the role of social support on participation in sporting activities. Furthermore, there is no evidence of the association of psychological adjustment with sports activities, and no previous studies have explored the role of social support on psychological adjustment in sports activities. These are significant gaps in the literature that led us to examine the role of social support as a psychosocial factor associated with sports activities. Addressing the national priority for enhanced fitness and physical well-being requires a comprehensive understanding of the impact of social support on the participation of Muslim women in sporting activities. The results of this study may shed light on the level of social support available for Muslim women, the impact of psychological adjustment on sports activities, and the relationship between psychological adjustment and sports participation. Additionally, the results of this study may support and further develop the Social Ecological Theory and SCT, enrich horizontal data studies of the sporting participation of Muslim women, promote the benign development of the national relation, and provide effective consultation services at the level of decision-making for relevant management work of the government.

## 2 Literature review and hypothesis research

### 2.1 Social support and sporting activities of Muslim women

Social support refers to the support and assistance received from families, friends, colleagues, and neighbors, among others, including practical and emotional support (Huang et al., 2017). Practically, social support can effectively relieve daily mental stress and improve the individual’s ability for social adaptation (Cho et al., 2023). Xiao (1994) believes that social support includes objective support, subjectively perceived social support, and utility of support.

A Chinese survey of female health has shown that more than 60% of women take the initiative to do something positive for their health; however, only 25.8% of women are able to train three times a week. This suggests that women are influenced by their social situation (such as work, family, and marital status) (Xiong, 2020). Results of the survey also indicate that there is a gender-based labor division in society (such as the role of carer and observer). Other elements of social support also influence participation in sporting activities. Islam advocates a healthy lifestyle and encourages both men and women to participate in sporting activities to maintain a healthy physical state, and it only restricts the standardization of costumes and separates activities from men (Bibi et al., 2016). However, due to their misunderstanding of the religion and a lack of knowledge of religious affairs, most Muslim women do not participate in sporting activities, worrying that participation in sporting activities may lead to a negative view of their beliefs and social and cultural demands (Yasmeen, 2011). However, Muslim women of the Hui nationality in the central region of China are very active in sporting activities, including martial arts, walking races, square dancing, and ball games. Sports in general exhibit similarities to the activities practiced by the local Han population (Wang et al., 2018).

Research has revealed that social support significantly influences participation in sporting activities, with high levels of social support effectively boosting participation by Muslim women in such activities (Banerjee Landry et al., 2017). Research on social support for Muslim women has been mainly conducted from three dimensions:

First, social support for Muslim women is precious. It functions as a comprehensive network that connects individuals, thus offering both practical and emotional support and facilitating closer connections and access to social resources. This support is primarily evident in geographical ties, affinity, and other close relationships (Chang, 2014). Therefore, stronger societal relationships among urban Muslim residents can lead to increased social support regarding employment assistance and leisure activities (Li and Yang, 2015). Sufficient domestic support can distinctly improve the frequency of participation by Muslim female teenagers in sporting activities (Mukherjee and Khatun, 2022). Support from husbands and relatives with information can promote participation by pregnant women in sporting activities (Yang and Guo, 2020) and their participation frequency in aerobic exercise (Keller et al., 2014).

Second, support from the social environment for Muslim women involves providing environmental opportunities for participation in specific behaviors by setting standards and implementing social control measures to reduce pressure and influence personal behavior (Cañas et al., 2019). The social environment can influence individuals’ self-esteem in the working, domestic, and institutional environments. Support for Muslim women in the social environment includes cultural acceptance and recognition. When Muslim women overcome the potential barriers of cultures and religions, they may develop a more positive attitude toward participation in sporting activities (Aljayyousi et al., 2019). Equally, the provision of adequate facilities and resources is important. Studies have shown that Muslim women are more likely to participate in sports when they have access to dedicated sports facilities and opportunities that address their specific needs and preferences (Thorpe Ahmad et al., 2022). The last element of social support is education and awareness. By enhancing society’s understanding of gender and cultural diversity, prejudices can be mitigated, leading to increased opportunities for Muslim women to engage in sporting activities (Abdulwasi Bhardwaj et al., 2018).

Third, support for the social beliefs of Muslim women refers to the emotional, anticipatory, and social support offered and received by the individuals in their religious beliefs groups. This type of support emphasizes the emotion of a common belief, social justice, and tangible interpersonal relationships (Xiong, 2021). Research suggests that during the praying process, the priest can impart advice on beneficial and healthy activities to women, which motivates women to participate in sporting activities. Furthermore, supporting the social belief received later can inspire women to participate in sporting activities (Eldoumi and Gates, 2019). Regular discussions about sporting activities among church members can foster encouragement and mutual support, motivating women to participate in sporting activities together (Kegler Escoffery et al., 2012). In addition, research on Muslim women in China predominantly employs inferential methods and interviews, with few studies using cross-sectional approaches.

In short, Muslim women’s sporting activities are refined, mainly influenced by traditional concepts and religious beliefs. If the society holds a positive and inclusive attitude toward the participation of Muslim women in sporting activities; provides corresponding supporting resources of institutions, organizations, and policies, for example; and offers opportunities and chances for women to participate in sporting activities, their willingness and ability to participate in sporting activities can be considerably improved. Therefore, research hypothesis H1 was proposed:

*H1*: Social support has a positive influence on the level of participation in sporting activities by Muslim women.

### 2.2 Social support and psychological adjustment

Psychological adjustment involves the application of psychological science to regulate emotions, willpower, intentions, and other psychological activities aimed at maintaining or restoring a normal psychological state. It can be undertaken by individuals themselves or with the assistance of others (Wang, 2014). Traditional psychology emphasizes the function of restoration within a psychological adjustment. The adjustment mode has been described as the passive reaction to a stimulus (Dufner et al., 2019). Positive psychology highlights the humanized and positive aspects of psychological adjustment and believes that humanity has a particular tendency and potential for obstacle resistance, which, as it increases, helps to overcome unhealthy factors and lays the foundation for generating positive subjective experiences and fostering an upbeat personality. It is also crucial in cultivating an individual’s adaptive capacity. The target of psychological adjustment is not to repair trauma but to develop and cultivate it to develop a strong personality. The process of psychological adjustment does not cure patients, but instead, it fosters positive adaptation to the environment by encouraging individuals to explore and build upon their strengths (Zheng et al., 2017).

Research has shown that social support received by married Muslim women can be conducive to improving their living quality and that their degree of satisfaction toward life is also a positive correlative factor. The person who accepts her need for affection often has a favorable health and mental status (Aizize et al., 2014). Studies on Muslim medical students have revealed a strong correlation between social support and various forms of adaptability, including interpersonal, emotional, and self-adaptability. This correlation is evidenced by the relationship between social support and adaptability within the context of school. In addition, domestic support correlates with adaptability to the school (Kader et al., 2011). Khatib Ben-David et al.’s (2021) research indicates that social support has a significant and positive influence on widowed Muslim women’s growth after suffering from trauma. Additionally, social support is crucial in fostering an individual’s psychological development and cultivating self-esteem, and it can potentially enhance overall psychological well-being (He et al., 2021). Since common beliefs, national religions, and cultures are shared, urban Muslims develop a particular social network that becomes their primary source of support in their hometown and a place where they turn for psychological consolation (Chang, 2014). Social support acts as a buffer against mental stressors, indirectly protecting health while also directly maintaining positive emotional experiences and mental states. This fosters a balanced mindset, which is essential for overall health (Zheng et al., 2009). On this basis, more support—such as emotional support and support for recognition of their identity—can help Muslim women relieve their anxiety and aloneness, stabilize their emotions, provide greater social support, enable them to cope with challenges and hardships in life calmly, recover from hardships, and maintain a high level of psychological adjustment. Therefore, research hypothesis H2 was proposed:

*H2*: Social support has a positive influence on the level of Muslim women’s psychological adjustment.

### 2.3 Psychological adjustment and sporting activities

Some studies have indicated that positive emotions during the women’s physiological cycle can improve the continuity of sports (Prado Silveira et al., 2021). Social anxiety and aloneness can influence teenagers’ participation in sports (Brière Yale-Soulière et al., 2018). Through an intervention study of 44 women and their behavior in sports, Wilson et al. (2003) proposed that the function of psychological adjustment has a positive effect on the promotion of sports exercises. A positive mental state can promote primary school students’ enthusiasm for attending physical education classes. Students’ learning effects from physical education classes can be improved by the psychological drive of their enthusiasm (Jiang, 2023), and positive psychological adjustment can result in students performing at a higher level (Brière Yale-Soulière et al., 2018). In an ideal psychological adjustment process, individuals continuously aim to improve themselves, fostering positive self-construction and growth (Hepper Gramzow and Sedikides, 2010). Hence, effective psychological adjustment can encourage engagement in sporting activities, resulting in a psychological state significantly superior to average. This has profound implications for individuals, motivating them to actively participate in sports for both physical health and mental well-being (Zhou et al., 2022). Based on these studies, research hypothesis H3 was proposed:

*H3*: Psychological adjustment has a positive influence on participation in sporting activities.

### 2.4 Mediating effect of psychological adjustment in social support and sporting activities

While there is a lack of studies on the mediating effect of psychological adjustment between social support and sporting activities, some studies have identified partial relationships between these factors. Psychological adjustment often mediates the relationship between external support and individual behavior. In his study on teenagers, Xiang (2014) proposed that support for autonomy in sports can influence teenagers’ satisfaction with being cared for by others and their understanding of psychological demands, which can further influence their level of extracurricular sports. Psychological adjustment often plays a mediating role in studies investigating the influence of social support on individual behavior. Additionally, one study has shown that improving social support results in an improvement in high-level demands, further improving the level of psychological adjustment (Yu and Sun, 2021). Therefore, individuals are more dedicated to their work. Previous research has shown that psychological adjustment can play a mediating role in the influence of social support on independent health evaluations (Gao et al., 2022). Bolis et al. (2023) indicated that increased social interactions and emotional well-being can alleviate mental pressure, foster optimism, enhance psychological adjustment, and improve individual health. Studies investigating the influence of social support on individual behavior, such as dedication at work (Yu and Sun, 2021) and self-evaluated health (Gao et al., 2022), have found that psychological adjustment often plays a mediating role. Based on the above studies, it can be inferred that psychological adjustment in Muslim women mediates the relationship between social support and sporting activities—more robust social support correlates with higher psychological adjustment, which leads to increased physical activity levels. Therefore, research hypothesis H4 was proposed:

*H4*: Psychological adjustment plays a mediating role in the relationship between social support and participation in sporting activities.

**Figure 1:**
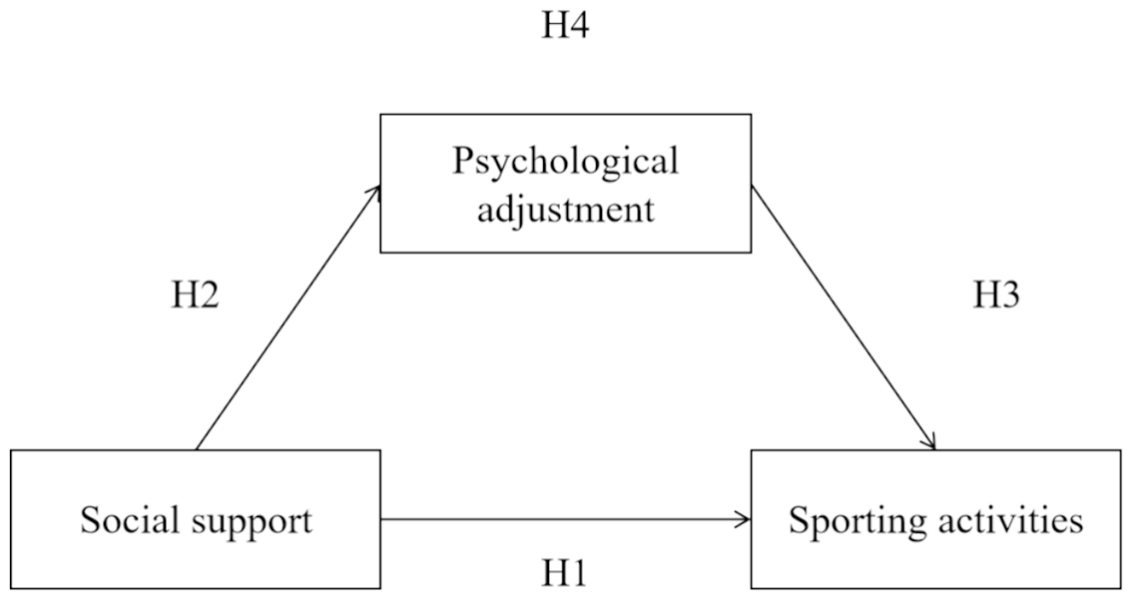
Hypothesis model.

Psychological adjustment is pivotal in promoting the participation of Muslim women in sporting activities. Nevertheless, there is a need for further studies on Chinese women with Islamic beliefs. Given this situation, by taking social support as guidance and psychological adjustment as the entry point, the theme was combined with localized studies to establish the path model of Muslim women’s participation in sporting activities. The aim of the study was to investigate the relationship between psychological adjustment in Muslim women and the effect of social support and sporting activities. The model of the research hypotheses is shown in Figure 1.

## 3 Research methods

### 3.1 Ethical statement

This study was approved by the Ethics Committee of Anyang City Third People’s Hospital (Approval No. 20220063). Participants were selected using convenience sampling technique and provided informed consent, which was reviewed by the Ethics Committee of Anyang City Third People’s Hospital. The study conformed to the ethical standards in the 1964 Declaration of Helsinki.

### 3.2 Research sample

The study focused on Muslim women residing in areas predominantly inhabited by the Hui people in central China. A questionnaire survey was conducted among Muslim women living in Zhengzhou, Zhoukou, Luoyang, and Kaifeng mosques. Convenient sampling methods were employed to select participants. Researchers enlisted mosque imams to recruit Muslim women for questionnaire completion at the mosque and collaborated with the Martial Arts Association of Hui Nationality and its square-dancing teams to maximize the number of participants. Instructions were provided and questionnaires were collected on site. A total of 409 questionnaires were distributed, and 336 were collected (82%). There were 35 invalid and 301 valid questionnaires (91%). Demographic data of respondents are shown in Table 1.

**Table 1:** Population demographic data.

Variable	Category	Number of people	Percentage
Age	Under 18 years old	11	3.7%
	19–29 years old	7	2.3%
	30–39 years old	28	9.3%
	40–49 years old	49	16.3%
	50–59 years old	94	31.2%
	60–69 years old	74	24.6%
	>70 years old	38	12.6%
Education background	Junior high school and below	156	51.8%
	Senior middle school and above	145	48.2%
Number of surrounding people participating in sporting activities	0–1 person	92	30.6%
	2 people	62	20.6%
	≥3 people	147	48.8%
Participated event	Martial arts/Tai chi	122	40.5%
	Walking/jogging	99	32.9%
	Square dancing/catwalk	67	22.3%
	Other events	14	4.3%

### 3.3 Measuring tool

#### 3.3.1 Measurement of social support

The Social Support Rating Scale formulated by Xiao (1994) was adopted to investigate the social support for the “empty-nest” elderly population. The scale included three dimensions: subjective support, objective support, and support utilization, involving a total of 10 items. Cronbach’s α coefficient in the questionnaire of this study was 0.756, KMO = 0.689, and the factor load of various questions ranged between 0.549 and 0.950. The fit index of the confirmatory factor analysis was χ^2^/df = 1.93, RMSEA = 0.056, CFI = 0.982, NFI = 0.964, IFI = 0.982, GFI = 0.974, and TLI = 0.970 (Table 2). Wu (2011) claimed that the ratio of χ^2^/df in the structural equation model construction and confirmatory factor analysis was between 1 and 3. The value of RMSEA was <0.08. CFI, NFI, IFI, GFI, and TLI values >0.9 suggested a good fit between the data and the model.

**Table 2:** Reliability and convergent validity of the study construction.

Construct	Item	Standard loading	S.E.	C.R.	P	CR	AVE	Cronbach’α
Subjective support	Social support1	0.549						
	Social support 3	0.865	0.177	9.62	***	0.821	0.615	0.803
	Social support 4	0.891	0.183	9.569	***			
Objective support	Social support 6	0.781						
	Social support 7	0.950	0.398	3.076	0.002	0.860	0.756	0.852
Utilization of support	Social support 8	0.785						
	Social support 9	0.737	0.098	9.779	***	0.756	0.511	0.749
	Social support 10	0.611	0.089	8.895	***			
Toughness	Resilience 11	0.741						
	Resilience 16	0.843	0.081	14.311	***	0.864	0.614	0.860
	Resilience 17	0.836	0.076	14.205	***			
	Resilience 18	0.706	0.080	11.935	***			
Self-improvement	Resilience 5	0.820						
	Resilience 7	0.805	0.061	15.521	***	0.857	0.601	0.855
	Resilience 8	0.717	0.067	13.346	***			
	Resilience 24	0.754	0.064	14.252	***			
Optimism	Resilience 4	0.752						
	Resilience 6	0.831	0.082	13.296	***	0.791	0.560	0.784
	Resilience 2	0.650	0.076	10.622	***			

The symbol ***indicates *P* < 0.001.

#### 3.3.2 Measurement of psychological adjustment

Psychological adjustment is composed of resilience, forgiveness, and self-compassion (Curtis Danquah and Aunger, 2009). In this study, it was observed that the scales of forgiveness and self-compassion were incompatible with the population of Muslim women. Therefore, the psychological resilience scale was the only measure adopted to assess psychological adjustment. The study used the Connor-Davidson Resilience Scale (CD-RISC) developed by Conner and Davidson in 2003. Yu and Zhang translated, revised, and published its Chinese version in 2007 (Yu and Zhang, 2007). Currently, this scale is the most widely used scale of psychological resilience in China. It includes 25 items and three dimensions: toughness, self-improvement, and optimism. The study used a Likert five-point scale method, offering five options: “totally not conform,” “not conform,” “mediocre,” “conform,” and “totally conform,” corresponding to points 1 through 5 for each question. Respondents with higher scores were considered to have greater psychological resilience. Cronbach’s α coefficient in the questionnaire of this study was 0.920, KMO = 0.922, and the factor load of various questions was between 0.650 and 0.843. The confirmatory factor analysis yielded the following results: χ^2^/df = 2.724, RMSEA = 0.076, CFI = 0.962, NFI = 0.941, IFI = 0.962, GFI = 0.936, TLI = 0.948. These values met the model’s fitting standard (Table 2).

#### 3.3.3 Measurement of participation in sporting activities

To measure participation in sporting activities, a method similar to that used by Tan et al. (2020) was adopted. This method comprised three factors, namely frequency of participation, time of participation, and duration of participation. Each factor included five options, corresponding to 1–5 points.

### 3.4 Statistical method

SPSS24.0 and Amos24.0 were used for statistical analysis. Invalid questionnaires were excluded, valid data were processed, and corresponding scores were calculated. First, exploratory factor analysis and confirmatory factor analysis of the modified Scale of Social Support and the Scale of Psychological Resilience were conducted. The main components of various scales were extracted, and the reliability and validity of the questionnaire were investigated. Then, Pearson’s correlation analysis and the PROCESS macro developed by Hayes (2012) were conducted. The simple and moderated mediation models were tested using the built-in Model 4 in PROCESS (Hayes, 2012). The results are presented in Tables 3, 4. Furthermore, the Bootstrap method of deviation correction percentile was used to estimate 95% of the confidence interval (CI) of the mediating effect with 200 times sample sampling. The fit index χ^2^/df was <0.8, and the value of RMSEA was <0.08, while values of CFI, NFI, IFI, GFI, and TLI were all >0.9. A value of *p* < 0.05 indicated statistical significance.

**Table 3:** Descriptive statistical analysis and correlation coefficient of various variables.

	M ± SD	SS	OS	US	Toughness	SI	Optimism	Frequency	Duration	Time
SS	3.13 ± 0.82	1								
OS	2.41 ± 1.35	0.110	1							
US	2.69 ± 0.88	0.420^**^	0.151 ^**^	1						
Toughness	3.90 ± 0.82	0.278^**^	0.253 ^**^	. 288 ^**^	1					
SI	3.91 ± 0.84	0.341^**^	0.197 ^**^	. 304 ^**^	0.729 ^**^	1				
Optimism	3.97 ± 0.82	0.349^**^	0.207 ^**^	. 315 ^**^	0.646^**^	0.678^**^	1			
Frequency	4.17 ± 1.18	0.360^**^	0.173 ^**^	. 266 ^**^	0.288 ^**^	0.329 ^**^	0.331 ^**^	1		
Duration	3.50 ± 1.46	0.299^**^	0.153 ^**^	. 254 ^**^	0.245^**^	0.264 ^**^	0.238 ^**^	0.490^**^	1	
Time	3.64 ± 1.30	0.284^**^	0.239 ^**^	. 320 ^**^	0.333^**^	0.330^**^	0.304^**^	0.585^**^	0.488^**^	1

*Means *p* < 0.05, and ** means *p* < 0.01. SS, subjective support; OS, objective support; US, utilization of support; SI, self-improvement; Frequency, frequency of participation; Duration, duration of participation; and Time, time of participation.

**Table 4:** Mediating effect of psychological adjustment between social support and sporting activities.

Outcome variable	Predictor variable	R	R^2^	F	*β*	Se	T
Psychological adjustment	Social support	0.405	0.203	76.281^***^	0.552	0.063	8.733^***^
Sporting activities	Social support	0.501	0.251	50.060^***^	0.606	0.082	6.037^***^
	Psychological adjustment				0.361	0.082	4.400^***^

The symbol ***indicates *P* < 0.001.

## 4 Results and analysis

### 4.1 Control and test of common method bias

Regarding the actual survey procedure, all questionnaires were filled in anonymously, and regular integrals and inversion integrals were used to control potential method bias. The Harman single-factor test method was adopted for verification. The KMO test and Bartlett’s sphericity test were conducted for the data, and the results showed that KMO = 0.881, Bartlett value = 3335.217, df = 231, and *p* < 0.001, indicating that the data were suitable for factor analysis. All variables were included in the analysis of exploratory factors not rotated—additionally, five factors with characteristic roots that were more significant than one were extracted. The maximum factor’s variance explained 27.43% of the variance, below the critical 40%. This indicated the study’s absence of significant standard method bias, which made it suitable for further analysis.

### 4.2 Correlation analysis

Pearson’s Product Moment correlation was employed for analysis (Table 3). With the exception of subjective support and objective support, which were not significantly correlated, a positive correlation (*p* < 0.05) was evident between all variables, which suggests a relatively high degree of connection between social support, psychological adjustment, and sporting activities of Hui women.

### 4.3 Mediation analysis

The plug-in PROCESS in the software SPSS24.0 programmed by Hayes (2012) was adopted, and Model 4 was employed. Social support was considered the independent variable, psychological adjustment was the mediating variable, and sports activity was the dependent variable. The mediating effect was assessed using Bootstrap analysis with 5,000 Bootstrap samples and a 95% CI significance level. A parameter in the model was considered significant if its CI did not include 0.

The result showed (Tables 4, 5) that social support had a significant predictive effect on sporting activities (*β* = 0.805, *p* < 0.01), and its direct predictive effect on sporting activities remained significant (*β* = 0.606, *p* < 0.01) when mediating variables were added. Meanwhile, social support exerted a significant predictive effect on psychological adjustment (*β* = 0.199, *p* < 0.01), while psychological adjustment also had a significant predictive effect on sporting activities (*β* = 0.361, *p* < 0.01).

**Table 5:** Decomposition table of total effect, direct effect, and intermediary effect.

	Effect	BootSE	t	BootLLCI	BootULCI	Relative effect size
Total effect	0.805	0.0924	8.722^***^	0.624	0.987	
Direct effect	0.606	0.1	6.037^***^	0.408	0.804	75.30%
Indirect effect	0.1993	0.059		0.093	0.323	24.70%

The symbol ***indicates *P* < 0.001.

In addition, the direct effect of social support on sporting activities, namely the upper and lower limits of 95% of the CI of Bootstrap of the mediating effect focusing on control, did not contain 0 (Table 5). This indicates that social support could directly predict sporting activities through the mediating effect of psychological adjustment. Herein, the direct effect (0.606) and mediating effect (0.199) accounted for 75.3 and 24.7% of the total effect, respectively.

## 5 Discussion

### 5.1 Effect of social support on Muslim women’ sporting activities

As indicated by the structural model, social support for Muslim women significantly and positively influenced their participation in sporting activities, thereby supporting H1. The study results are consistent with previous literature (Banerjee Landry et al., 2017; Mai and Shi, 2019; Pan et al., 2020). One study of college students demonstrated a significant correlation between the behavior and form of exercises and social support. Moreover, increased social support was found to correlate with greater engagement in sporting activities (Cho et al., 2023). It has also been suggested that support should be provided for college students in four aspects: sports skills, sports time, sports environment, and sports publicity (Zhu and Dong, 2018). The level of domestic support is the critical factor influencing the participation of Muslim women in fitness clubs. Examples include the environment, financial resources, logistical support, emotional encouragement, and parental influence, all of which positively correlate with participation in sporting activities (Timperio et al., 2013). Social support from family members has been found to significantly increase the frequency of Muslim adolescent female participation in physical activities (Mukherjee and Khatun, 2022). This indicates that assistance and support can encourage Muslim women to participate in sporting activities and form stable, active, and lasting awareness of sporting participation.

Social Ecological Theory emphasizes that a multi-layered social support system is essential for the health and development of the individual. Social Ecological Theory explains how social support influences sports participation behavior at different levels. At the individual level, social support enhances motivation and ability to participate through the provision of emotional incentives and practical help. At the family and friends level, encouragement and companionship increase the frequency and persistence of physical activity. At the organizational level, the facilities and supportive culture provided by schools, workplaces, and community-based organizations encourage participation in sports (Luan et al., 2023). Support at community and policy levels is equally important. Community health clubs, public facilities, and community events provide easy access to participation opportunities and enhance community cohesion. Additionally, government and NGO sports policies and programs promote individual and community physical activity through systematic support. Multi-level social support systems working together can significantly enhance sports participation behaviors (Li et al., 2024).

With sporting activities, there is a positive relationship between increased social support and better self-esteem protection. Social support enables individuals to effectively cope with challenges with a positive mindset, enhancing their confidence and cognitive abilities. Muslim women have a more positive attitude toward sporting participation when they overcome potential cultural and religious barriers (Aljayyousi et al., 2019; Miao et al., 2020). Participation in sporting activities increases when independent sports venues are provided for Muslim women (Thorpe Ahmad et al., 2022). Frequent discussion, encouragement, and companionship among church members can also increase the frequency of physical activity (Kegler Escoffery et al., 2012). Thus, religious management departments and communities can play a significant role in shaping Muslim women’s personal beliefs, daily practices, and lives. For example, mosque imams can promote beneficial and healthy sports activities among female believers by helping to raise awareness about the health benefits of exercise. They can integrate sports activities into daily life, create dedicated exercise time, provide necessary infrastructure, enhance the sporting environment, and expand the dedicated spaces for sporting activities for Muslim women (Fan et al., 2019; Thorpe Ahmad et al., 2022).

### 5.2 Mediating effect of psychological adjustment in social support and Muslim women’s sporting activities

The study results confirmed the significant and positive influence of social support on the psychological resilience of Muslim women, supporting H2. In their study, He et al. (2020) found that the correlation coefficient between social support and psychological resilience was 0.360. The higher the level of social support the individual perceives, the stronger the individual’s power to mobilize resources to reach a reasonable state to cope with stress and difficult situations (Shi et al., 2019). Married Muslim women benefit from social support, which enhances their quality of life and improves their physical and mental health (Aizize et al., 2014). The perceived social support of Muslim medical students can significantly improve their interpersonal and emotional adjustment in school (Kader et al., 2011). Social support also has a significant positive impact on post-traumatic recovery in Muslim widows (Khatib Ben-David et al., 2021). A study of students in Islamic boarding schools also revealed that the social support offered by companions significantly influences self-adjustment (Ghorbani et al., 2019), and students who obtain social support can quickly adapt to the school’s environment, which can improve their psychological adjustment capacity. In general, emotional support fosters emotional well-being and a sense of care, which ensures that individuals receive assistance and emotional validation when needed. It helps to create a safe and inclusive environment, encouraging emotional expression. Containment and openness can make individuals share their concerns, express feelings, and win empathy. Emotional support can help individuals cope with emotions, which strengthens the overall sense of happiness (Acoba, 2024). Hence, as Muslim women perceive support from families, friends, and religious groups, they also build a psychological buffer of adjustment. With high levels of support, they are more inclined to protect themselves and their social networks, resisting the impact of adverse factors during life or environmental changes.

In this study, the equation model demonstrated the significance of psychological adjustment in influencing participation in sporting activities, thus supporting H3 and aligning with previous studies (Hannan et al., 2015; Eskandarnejad, 2018; Huang, 2024). Psychological resilience is a type of composite psychological resource, which is a combination of attitude, emotion, and behavior (Dufner et al., 2019). According to SCT, individuals with higher psychological resilience possess psychological resources, such as optimism and vitality, along with a high degree of psychological flexibility. Consequently, the level of their participation in sporting activities may be enhanced accordingly (Bandura, 1978). Individuals with high psychological resilience also have better self-regulation, a stronger will to target achievement, and stronger willpower and action perseverance (Carriedo Cecchini et al., 2020). During exercise, individuals with a determined mindset and a positive attitude have a higher threshold for physiological limits. By employing self-regulation strategies, they can optimize their sporting experience, leading to satisfaction and happiness, which enhances their motivation for participation in sporting activities (Zhou et al., 2022).

Herein, psychological adjustment was found to partially mediate the effect of social support on participation in sporting activities, with the mediating effect accounting for 26.5%. This supports H4 and also indicates that social support for Muslim women has a direct influence on their participation in sporting activities, and it could also generate indirect influences by psychological adjustment. Zhang et al. (2021) suggested that psychological resilience may have a mediating effect on the process by which college students learn how to cope with life’s challenges with the help of social support, a finding similar to the result of this study. Higher perceived social support has been found to correspond to greater psychological resilience, enabling individuals to confront life’s challenges positively and effectively with coping strategies (Shi et al., 2019). Psychological resilience aids the individual’s mobilization of more resources (such as cognitive resources), thus enabling them to quickly adapt to the constantly changing environment (Niu et al., 2016). As an external protective factor, social support is essential for the participation of Muslim women in sporting activities.

The dynamic model of psychological resilience suggests that individuals’ needs for safety, belonging, love, and respect should be met during their upbringing. Material and emotional support from families, companions, and society are vital resources for fulfilling these needs. If external protective factors meet individuals’ psychological needs, they will be able to use these tools, improve their psychological resilience, and further promote the development of their actions related to physical health (Yan, 2021). Hence, individuals with high levels of psychological resilience exhibit a stronger determination to follow through with their exercise intentions, making psychological resilience a reliable predictor of their actual exercise levels. Psychological resilience can reduce the negative influence of external adverse factors on individuals’ mental state and their ability to adapt (Sharifi et al., 2023). Therefore, domestic religious management departments should prioritize the cultivation and investigation into the role of psychological adjustment among Muslim women.

### 5.3 Research significance and theoretical contribution

Overall, this study enriches and expands the application of the theoretical models of psychology and sporting activity promotion and enriches the contents of SCT and Social Ecological Theory. However, different regions have different religious cultures. Chinese studies have mainly focused on Muslim women in cities in the northwest region of China (Su, 2018), and the influence of social support on participation in sporting activities has rarely been discussed from the perspective of Muslim women in central China. To this end, this study emphasizes the importance of social support for the participation of Muslim women in sporting activities. It also highlights the mediating effect of psychological adjustment, which has significant implications for policies on ethnic minority management, fostering the development of urban ethnic relations and promoting the integration of the Muslim population into cities and society.

Future efforts should focus on enhancing Muslim women’s perception of social support, encouraging community and religious organizations to prioritize domestic relations and psychological well-being, promoting awareness of scientific sports practices through education and outreach, and organizing sporting activities for women in a structured and targeted manner.

### 5.4 Limitations and future research directions

However, this study also has some limitations. First, due to the situation limitations, the convenience sampling method was adopted to collect 301 samples from Henan Province. The Muslim women involved in the samples were generally older, and there were few samples of younger Muslim women collected, showing an imbalance in age among the samples. In future research, differences in survey samples should be considered. The number of samples should be increased, and the scope of research should also be expanded to improve the value of related research. Second, self-reported questionnaires were distributed to collect data; however, the limitations of a cross-sectional study make the causal relationship between variables unreliable. Additionally, the study only discussed the predictive result based on relevant studies conducted in the early stage, and further long-term studies are required. Finally, the study confirmed the relationship between social support and participation by Muslim women in sporting activities, with psychological adjustment mediating this connection. The influence of a healthy mental state on sports participation was multifaceted, involving cognitive aspects and basic psychological needs. Further in-depth investigation into these factors is still needed.

Social integration, adaptation to urban environments, and overall living quality of Muslim women are important research topics and challenges that management departments should address. Exploring how sporting activities can contribute more effectively to social integration and improve the quality of life for Muslim women, as well as investigating the underlying mechanisms, are promising avenues for future research.

## 6 Conclusion

Social support significantly and positively impacts the psychological adjustment of Muslim women, underscoring its positive role in fostering psychological well-being. Additionally, social support positively influences participation in sports activities, suggesting that enhancing support systems can elevate the participation of Muslim women in sports. Meanwhile, the level of psychological adjustment significantly and positively influences the level of participation in sporting activities for Muslim women. In other words, the perception of social support by Muslim women can directly influence their participation in sporting activities, along with improving their psychological adjustment level.

## Data Availability

The original contributions presented in the study are included in the article/supplementary material, further inquiries can be directed to the corresponding author.
